# Syphilis of Upper Air Passages

**Published:** 1904-10-29

**Authors:** 


### SYPHILIS OF UPPER AIR PASSAGES.

**Syphilis.** — St. Clair Thomson[1] has discussed syphilitic affections of the upper air passages and their treatment. Prompt recognition in these affections may be of vital importance, as they progress sometimes with alarming rapidity. The possibility of an obscure neoplasm or thickening in the nose, pharynx, or larynx being of syphilitic origin must always be borne in mind. Primary chancre may be met with in the vestibule of the nose, arising from inoculation with the finger nail. Tonsillotomy has been followed by a chancre on the tonsil. Secondary syphilis of the pharynx chiefly assumes the form of erythema, or of mucous patches, and these manifestations last from a few weeks to some months. Mucous patches are apt to recur in the tertiary period. Erythema should be looked for by daylight rather than by artificial light. A gumma may form in the trachea and escape notice, and be followed by tracheal stenosis. General hygienic treatment is very important, including that with abundance of fresh air. Secondary syphilitic affections of the nose and throat generally respond quickly to iodide of potassium taken internally, with or without mercury. The author recommends the inunction of mercury in all severe and intractable cases of tertiary disease of the throat. The drug, given in this way, seems to have the power of causing the resorption of gummatous infiltration, and thus preventing contractions from scar tissue. It is not loss of the septum which causes the saddle-back nose, but disease of the lateral arches or cicatrical contraction. Iodide of potassium often fails to prevent cicatricial contraction. When iodides are badly borne, syrup of hydriodic acid (Gardner) may be effective. Cicatricial deposit in the larynx is apt to cause permanent impairment of voice, and a post-syphilitic tendency to hypertrophic growth. In sloughy inflammation of the throat the inhalation of sublimed calomel is useful, and the dose may gradually be increased from 3 to 15 or 20 grains. The best local treatment for mucous patches in the pharynx is with caustics—nitrate of silver fused on a probe, acid nitrate of mercury on a match stick, chromic acid solution in cotton wool (20 to 30 grains to 1℥). An excellent gargle is composed of chlorate of potash, 10 grains, black wash 1℥ and water 1℥. Peroxide of hydrogen is very useful in tertiary syphilis of the nose with ozæna; also antiseptic washes such as listerine, sanitas, or glycothymoline, followed by antiseptic powders—*e.g.* aristol, europhen, etc. In severe cases of ozæna, after cleansing and insufflating heated calomel, the nose may be packed with double-cyanide gauze as recommended by C. A. Parker.

[1] The Practitioner, July 1904, p. 118.

